# Hospital and Institutional News

**Published:** 1920-02-07

**Authors:** 


### HOSPITAL AND INSTITUTIONAL NEWS.

### The New Superintendent of Guy's Hospital.

FOLLOWING upon the retirement of Sir E. Cooper Perry, we have to welcome as the new superintendent of Guy's Hospital Mr. H. L. Eason, C.B., C.M.G., M.D., M.S., who brings with him to his new appointment a reputation and career of which both he and his hospital may be justly proud. Mr. Eason came to Guy's as a University of London student from University College, and graduated M.B. in 1899. All who knew him in those days will recall a truly live man; the intimate part he played in the activities of that happy band who originated and ran the first *Guyoscope*, a periodical the wit and brightness of which it was, and will ever be, uncommonly hard to beat. Another glimpse of those early days is given by the *Guy's Gazette*, which reminds us of his leadership among the "Dis-Guysed Minstrels," who were, we are told, in spite of the excellence of Christmas performances so recently witnessed, "a band who are to the Christmas-Day niggers what the performers in Italian comedy were to our friends of the streets, Mr. Punch and his Judy."

Equally distinguished in his profession, Mr. Eason was appointed house physician to Dr. Frederick Taylor in January 1900. In December 1901 he took his M.D.Lond., and only a year later the M.S. of the same university. Meanwhile, he had held the appointment of ophthalmic assistant to Mr. Brailey, and in 1902 commenced his administrative career as Warden of the College. In 1904 a precedent was created in that he became Dean of the medical school before his election to the full staff, but this followed in late 1905, when he succeeded Mr. Brailey as ophthalmic surgeon to the hospital, in which capacity the depth of his knowledge and his operative skill are by-words in modern Guy's.

The technical side still had but partial claim on his attention, and in 1912 he was elected to the Senate of the University of London, but his appointment as Consulting Ophthalmologist to the Forces in the Mediterranean in the September of 1915 caused his retirement, and in October of that year he arrived in Mudros. Later, after the evacuation at Gallipoli, he was transferred to Egypt in the same capacity to the Egyptian Expeditionary Force, and with these troops he remained until his return to this country in July 1919.

During his Army experience the value of Mr. Eason's work is particularly noteworthy. Those who were fated to visit the Eastern theatres at the middle period of the war know with what difficulty the man-power problem in Egypt was solved. One of the greatest factors in the success finally achieved was the provision of a uniform system of medical boards which could act in true accord with facts and requirements, and work alone and unhampered by the many irregularities rife at the time. In providing the required organisation Mr. Eason took a leading and praiseworthy part, and himself acted as President of the Standard Medical Boards for Classification and Invaliding in Alexandria. At the same period Mr. Eason was President of the Special Medical Board to the Flying Corps, and here, as elsewhere, he achieved a first-class record in both professional and administrative spheres. For these services he was made, first, C.M.G. in 1917, and C.B. in January 1919, and finally, purely ophthalmic work again calling for all his energy, he was found successfully combating an outbreak of ophthalmia among the Turkish prisoners resulting from Allenby's victory, a menace of indeed appalling rapidity and danger at the time.

So might one record many more achievements in a career which has deservedly consisted of one step after another to recognised and greater success. All who know him will agree that in their new superintendent Guy's have wisely chosen a man of action and resource, and we congratulate both the hospital and Mr. Eason upon this new appointment from which we foresee that Guy's, as a whole, will derive the greatest benefit.

### CONGRATULATIONS TO MR. BUCHANAN.

WE congratulate Mr. J. Courtney Buchanan on his appointment as secretary of the Cancer Hospital. He is a well-known figure in the hospital life of this country, and as honorary secretary of the British Hospitals' Association his name is familiar beyond the wide circle that has the pleasure of his acquaintance. Mr. Buchanan has twenty-four years' hospital service to his credit; he served nine years at St. Bartholomew's under Mr. Cross, then, after a comparatively brief stay at the Bolingbroke (during which the new buildings were erected), he proceeded to the Metropolitan Hospital, where he has been for nearly twelve years. During the war the Metropolitan took in adjacent buildings for the reception of wounded Service men, and the brunt of the very arduous administrative work entailed fell upon the shoulders of the secretary and house governor. Mr. Buchanan was educated at Christ's Hospital and left as a "Grecian," he became a student of Lincoln's Inn and was called to the Bar. The Cancer Hospital is fortunate in obtaining the services of an officer with such a record, and we do not think that the Metropolitan Hospital authorities are alive to present-day requirements when they seek to replace him by the offer of the very inadequate salary of £500 a year.

### THREE INTERESTING WILLS.

THE will of Mrs. Louise D'Este Oliver, of Collingham Gardens, S.W., sister of the late Lord Courtney of Penwith, is one in which a large number of people and institutions are mentioned. After leaving between £50,000 and £60,000 to relations, friends and servants, and a collection of pictures to the National Gallery, Mrs. Oliver provided for a number of charities. Amongst the chief bequests are £3,000 each to the Elizabeth Garrett Anderson Hospital and the West Cornwall Infirmary; £2,000 to the Dunedin Hospital, New Zealand; and £1,000 to the Cancer Charity of the Middlesex Hospital. Mr. David Shennan, a director of the Buenos Ayres Great Southern Railway, who left unsettled property of the gross value of £467,826, left £5,000 ("unless he shall have made such gift in his lifetime ") to the London Hospital, upon trust for investment in the discretion of the trustees of the hospital, and to apply the income for research work in connection with the septic form of Bright's disease. He also left £3,000 each to St. George's Hospital, the Victoria Hospital for Children, and the British Hospital, Buenos Ayres. Mr. Claud Watney, of the well-known brewing firm, left the greater part of his large fortune to his wife for life, with remainder to his children as she may appoint, or equally, "and failing issue who shall attain an interest," he left the reversion of the whole of his residuary estate, probably £500,000, to King Edward's Hospital Fund.

### INFLUENZA RETURNING.

Last week's circular from the Ministry of Health on the possibility of a new influenza wave, although given due notice in the Press, does not seem to have been regarded with any great seriousness by the public as a whole. Possibly this is due to the many warnings of last autumn which were followed by such a notable absence of the disease itself. The medical profession was at that time undoubtedly justified in preparing for a series of typical catarrhal outbreaks, and their patients should be thankful that their fears did not materialise. Nevertheless, the layman must not, as a result of this recent experience, fail to give due attention to the latest warning. On this occasion there is a simultaneous increase of true influenza in the great American cities, in Poland and also in the Far East, and although the latest returns for England and Wales fail to demonstrate that the infection has as yet reached this country, there is considerable probability of another wave developing at an early date. Of the precautions advised we would particularly draw attention to the ease with which prophylactic vaccination may now be obtained. "A vaccine against influenza has been prepared by the Ministry of Health and is available for general use in the same way as the War Office has provided similar vaccine for the troops. It is issued to Medical Officers of Health free of charge among medical practitioners within their districts, and any person who wishes to be vaccinated should apply to his private medical attendant. To obtain its value the vaccine should be used *before* the epidemic occurs. It cannot be guaranteed that the vaccine will necessarily protect from attack, but there is reason to expect that if an attack occurs vaccination will do much to lessen the risk of complications. Influenza is dangerous mostly because of what may follow it." There is no doubt whatever that within the last few days influenza—previously almost non-existent—has blazed up very definitely in London.

### UNIVERSITY OF MANCHESTER: CHAIR OF PHYSIOLOGY.

The Council has appointed Mr. A. V. Hill, O.B.E., M.A., F.R.S., to the Chair of Physiology, vacant through the resignation of Professor Stirling. Mr. Hill was a scholar of Trinity College, Cambridge, was third Wrangler in 1907, and was placed in the *first* class in Part II. of the Natural Science Tripos (Physiology) in 1909. He was George Henry Lewes's student, Walsingham, Gedge, and Rolleston Prizeman. In 1910 Mr. Hill was elected to a Fellowship of Trinity College, and in 1916 was elected a Fellow of King's College. In 1918 he became a Fellow of the Royal Society. During the war he obtained the rank of major in the Cambridgeshire Regiment and was appointed Director of the Anti-Aircraft Experimental Section of the Munitions Invention Department. The inventions which he made proved of the greatest value in the scheme of defence against attack from the air. His researches on the physiology of voluntary muscle are recognised as pioneer work of the first importance.

### THE REORGANISATION OF TEACHING AT UNIVERSITY COLLEGE HOSPITAL.

The system of medical and surgical "units," each under the control of a "director," for managing the branches of teaching and research at University College Hospital and school, has now come into full operation. In appointing Dr. T. R. Elliott, physician to the hospital, to be the first "director" ("professor" might be an equally suitable title) a most excellent choice has been made, for he is still a comparatively young man, has a very high scientific reputation, and has great gifts both for teaching and research. The details that have been published of the whole scheme show careful forethought and organisation; and they also seem to foreshadow a closer connection between hospital and school than has hitherto been customary—a development inevitable and necessary, but looked upon still with suspicion by some people of ultra-conservative tendencies. The director of the surgical unit is Mr. C. C. Chayce. An especially valuable feature of the scheme of teaching is the recognition of the recent great advances in the science of cardiology, contained in the proviso that during his course of duty under the director of the medical unit the student shall be attached for clinical clerking to special departments, such as the cardiographic department under Dr. Thomas Lewis.

### THE NEW MEDICAL PEER ON THE VOLUNTARY SYSTEM.

The voluntary hospitals may hope for a new spokesman now that Sir Bertrand Dawson has been raised to the peerage. If he is not a silent member of the Upper House (and why should he be?), his attitude can be divined from some remarks which he lately made at Birmingham to his medical colleagues of the Midlands. The big changes, he said, that were now coming over hospitals and the nation's provision for the sick must be adapted to foster the voluntary hospital tradition. The waiting-list of old time can no longer be tolerated; the number of patients must be expected greatly to increase. The needs are for more hospitals, their better distribution, and the better classification of institutions and of patients. Hospitals, like schools, must be within the reach of all. The expense of realising the dream will be heavy, but it will be the best investment, for, among other things, it will economise health. More beds mean prompt treatment, and prompt treatment is the beginning of prevention. Hospitals to-day are not only too few in number, but are called to do too much work, and much of it is the wrong work. We need grades of hospitals for different purposes. Public authority, he thinks, will have to support hospitals, but these must retain their voluntary workers, and State aid need mean only the appointment by the State of certain members of the committee. Penetration, not absorption, by the State is the clue, or it will be a bad thing for the community. Besides, patients do not object to pay. In primary hospitals a standard rate may obtain for public and private wards. The important thing is that both are wanted. The standard rate will include the cost of everything, except medical attendance. Patients will pay their doctors directly or indirectly, as before. But the rate must be elastic and in proportion to the patients' means. In the big hospitals and for the poorest the standard rate will include medical treatment, because the poor patient will not be followed to the hospital by his own doctor, but be in the charge of a consultant there.

### THE EARL OF CRAWFORD AS HOSPITAL HISTORIAN.

When the war started, the Earl of Crawford offered to the Red Cross Society his Lancashire house, Haigh Hall, at Wigan, and another known as The Woodlands. The Society accepted both and turned them into hospitals. That is now a matter of history, and fortunately the story has been written down under the title "Throughout the War," a volume which describes the history and the work of the Wigan and District Division of the British Red Cross Society. To this the Earl of Crawford has contributed an introduction, in which he makes an excellent point. "In England," he writes, "we developed the local hospital, with its own personality and character, to an extent unpractised by other belligerents." This is not only good criticism but good history. Local life has been at the root of the greatness of England. It is still congenial to her character, which it expresses; and just in so far as local life (a different thing from those central services which necessarily touch it) has been broken down before the Juggernaut of a central bureaucracy, just so far has national life and character decayed. The voluntary hospitals, among other things, are the guardians of this free tradition. Their independence and their voluntary character are its products. There is no better symbol of the permanent strain in English character than they. If they can keep the Ministry of Health in its proper place (and that is their duty) they will do a service to the nation too great for the nation to appreciate. That is why it is wiser, as Lord Crawford points out, to preserve the Voluntary Red Cross Organization than to lean upon Whitehall. If we can make the Ministry of Health largely unnecessary by the voluntary organisations which we have at hand, we shall have little to fear from it. And fear, in the best sense, is the patriotic attitude to adopt before every proposed increase of State departments, with the odious regimentation that it involves.

### THE LIFE OF HOSPITAL TRADITION.

Every hospital worthy of the name cherishes its own tradition, and that can only be kept fully alive by making every stone of the building a visible repository of the lives of the men and women who once worked there. Thus a ward-room can best be relieved of dulness by growing into a portrait gallery of its own great figures; a chapel should have memorial tablets; a hall its roll of chief officers; the very wards should contain as many named beds as possible, not because these have brought donations, but because every bed has thus associations of its own. Cottage hospitals, because they deal in small figures, are all the more to be commended when they too realise the value of what are often foolishly called "*æsthetic memorials*," as if an ugly name could disguise or extinguish a true and living thing. We therefore note with pleasure that Clacton Cottage Hospital has lately been presented with a portrait of the late Dr. W. H. Simon, J.P., who was its president for nearly twenty years. One good thing attracts another, and we therefore hope that in time this picture will be followed by others, and that the example of Clacton Cottage Hospital will be followed elsewhere.

### THE NEW HOSPITAL FOR MERTHYR.

Mr. E. M. G. Richards and Mr. C. M. Davies have been appointed architects to draw the plans for the new and greatly enlarged hospital proposed at Merthyr. This is to be, after all, a permanent hospital, since, on reflection, Mr. H. Seymour Berry has decided against the erection of a temporary hospital at Gurnos, and has agreed to expend the £10,000 which he promised towards the cost of an immediate building upon two wards, which are to be added to the existing general hospital. That now consists of forty-two beds. The proposed new hospital will accommodate 130, and towards the latter scheme for a permanent new building Mr. Berry has agreed to give £20,000 as well. He makes these generous gifts upon condition that a maintenance fund of £100,000 shall be raised. In the plans for the new hospital no assessor, we believe, is to have the casting vote, but Mr. Richards and Mr. Davies are to consult with the medical staff as the drafting of the plans proceeds. Unfortunately, most medical men know as little of architecture in relation to hospital design as architects know of medicine and hospital requirements; that is why an assessor or adviser, like

Dr. Mackintosh, of Glasgow, or an architect who has specialised in hospital design under such guidance, is an advantage that cannot be lightly dismissed.

### A MODEST INSTITUTION.

An unobtrusive London hospital, the Alexandra Hospital for Children, in Queen Square, has come into the open with an interesting appeal and a remarkable change of policy. After fifty years of unusually quiet work (it was founded in 1867) it has decided to move to the country, and to build on the Caversham hills a hospital of 150 beds for children, with 50 additional beds for men discharged from the services with tuberculosis of the bones and joints. It is primarily because the hospital treats tuberculosis that the change to the country is to be made. The change itself will reveal the existence of the hospital to many who had never heard of it before, because it is a long time since any appeal was issued. The patients found their way there easily enough; donors and subscribers have not been equally successful, though they would have been no less welcome than a tuberculous child. Luckily, however, the hospital has some resources. It has also received a conditional grant of £25,000 from the Red Cross. But it needs, when all is said, £50,000 more; and those who enjoy helping an old friend whose existence they had forgotten have now their opportunity to help in an unexpected quarter.

### A NEW RUNG IN THE HOSPITAL LADDER.

While the Lambeth Board of Guardians talks about providing hospital accommodation for special classes of patients, a new step is being taken at the Royal Free Hospital, which is at work to provide a new building for the use of paying patients of small means. Once again the voluntary system sets the pace! The new wards will be for men, for women, and for children. The site has been given. The plans, we believe, are prepared. The public is now being asked to provide the money. It should respond because one of the signs of the times has been the immense demand for admission to hospitals of patients who are willing and able to pay. Most nursing homes are nothing but the most extravagant kinds of hotels. Their fees are as ruinous as the houses which have been "adapted" as homes. Very few have been designed for their purpose. Very few, indeed, are not commercial in aim, and it is impossible, from their design and financial basis, that most of them should put the patient's interest before their desire to profit by him. Such a commercial failure cannot endure. Indeed, the best which have been built for their purpose, and designed to meet a need rather than to make a profit, are, in fact, small hospitals. But these are too few in number, and difficult to finance or to build. The proper place for the service which they supply on a small scale is a block for paying patients in connection with a voluntary hospital. The connection is good, is the best, for all parties. Design, finance, equipment, staff, are all there on their right basis. The profiteer and the sham "hospital" are removed at a stroke. The measure of public intelligence, therefore, can be gauged accurately by the success of the Royal Free Hospital's appeal.

### HOSPITAL BALLS AND SOCIAL LIFE.

Hospital fêtes in the Provinces are more interesting than those in London, because the former are leading social events in the district, whereas the latter, graced by whomsoever they may be, partake of the nature of a respectable, but amorphous, riot. In the same way London hospital dinners, bazaars, and dances are mere crushes, whereas in the Provinces people know their neighbours much as they do at a hunt ball. Thus while Provincial hospital dances, like hospital dinners in London, were equally interrupted by the war, the suspension of those in the country did something to rob Provincial life of its social amenities, and their resumption is important accordingly. A very sucessful ball was lately revived in connection with Warneford Hospital. The leading social lights of Leamington thronged the Town Hall for the occasion, the supper was a banquet, the lady patroness of the ball was the wife of the joint master of the Warwickshire Hounds, and herself late Commandant of the Kineton V.A.D. Hospital. We believe that the committee in charge has as much reason to be satisfied with the result as the dancers were with their programme, and the event has maintained its traditional claim to be the leading social gathering of the local season.

### FORTHCOMING LECTURES.

We would draw attention to the following Hunterian Lectures to be delivered in the theatre of the Royal College of Surgeons of England: "The Influence of Nerve Impulses on Gastro-intestinal Disorders—A Surgical Study," by Professor H. Tyrrell Gray, M.A., M.C., F.R.C.S., on Monday, February 9. "The Late Results of the Surgical Treatment of Chronic Ulcers of the Stomach and Duodenum," by Professor James Sherren, C.B.E., F.R.C.S., on Wednesday, February 11. Three lectures, "On the Historical Relationship between Experiments on Animals and the Development of Surgery," by Professor Walter G. Spencer, O.B.E., M.S., F.R.C.S.: I., on February 13; II., on February 16; and III., on February 18. The hour is in each case 5 o'clock P.M.

### THIS WEEK'S DRUG MARKET.

Business can hardly be described as active so far as the home trade is concerned, but the heavy demand from overseas tends to keep up prices, and most of the changes are in an upward direction. Cape aloes is in short supply and dearer, and ipecacuanha still tends upwards in price. Citric and tartaric acids continue to be in good demand, and both are quoted at higher rates. Cocaine seems likely to become dearer. Makers of glycerine have advanced their quotations. Essences of lemon and orange still tend to move upwards in price. Phenacetin, barbitone, aspirin, salicylic acid, salicylate of soda, and antifebrin are all dearer. Eucalyptus oil is in good demand, but supplies would appear to be ample.

